# Spatial Distribution and Temporal Trends of Dietary Niacin Intake in Chinese Residents ≥ 5 Years of Age between 1991 and 2018

**DOI:** 10.3390/nu15030638

**Published:** 2023-01-26

**Authors:** Li Li, Jing Sun, Huijun Wang, Yifei Ouyang, Jiguo Zhang, Tiantong Li, Yanli Wei, Weiyi Gong, Xuefei Zhou, Bing Zhang

**Affiliations:** National Institute for Nutrition and Health, Chinese Center for Disease Control and Prevention, Beijing 100050, China

**Keywords:** niacin, geographic information system, trends

## Abstract

Limited knowledge exists on trends in niacin consumption and the prevalence of inadequate intakes in China. Understanding trends and the spatial distribution of the prevalence of inadequate niacin intake is crucial to identifying high-risk areas and sub-populations. The dietary intakes of niacin between 1991 and 2018 were analyzed using the China Health and Nutrition Survey (CHNS) data. The estimated average requirement cut point was applied to estimate inadequacy. The geographic information system’s ordinary kriging method was used to estimate the spatial distribution of the prevalence of inadequate niacin intakes. However, between 1991 and 2018, the prevalence of inadequate niacin intake increased from 13.00% to 28.40% in females and from 17.75% to 29.46% in males. Additionally, the geographically significant clusters of high and low prevalence were identified and remained stable over almost three decades. The high prevalence of insufficient niacin intake was more pronounced in Henan and Shandong over 27 years. Further, effective and tailored nutrition interventions are required to address inadequate niacin intake in China.

## 1. Introduction

It is well known that vitamins play an important role in promoting health. The B vitamins serve as coenzymes in a vast array of catabolic and anabolic enzymatic reactions and are essential for oxidative processes. In addition, niacin deficiency results in pellagra, which was widespread in southern Europe and the United States during the 19th and early 20th centuries. This disease was subsequently eradicated by fortification. The evidence from the 2010–2013 China National Nutrition and Health Survey (CNNHS) showed that inadequate niacin intake remains common in China [1]. The effects of supplemental niacin and dietary intake of niacin on blood pressure, cardiovascular disease, blood lipids, and neuronal health have been assessed. Although study results have not always been consistent, increased attention has been paid to the effects of niacin on health [2,3]. One review speculated that optimal dietary intake of niacin may support neuronal health and delay neurodegeneration [4]. One study based on CHNS data showed an association between dietary niacin intake and hypertension. The study results showed that optimal dietary niacin intake might support the primary prevention of hypertension [5]. Higher intakes of niacin were associated with a reduced risk of metabolic syndrome [6].

In addition, several studies from different countries in various population sub-groups revealed geographical disparities in micronutrient intake. According to the 2010–2013 Chinese National Nutrition and Health Survey, geographical variation in the inadequacy of most nutrient intake was found in China [1]. In the United States, race and region were associated with the nutrient intakes of black and non-Hispanic white women [7]. However, sodium intake varied by geography, with the highest intake in the central region and among people living in rural areas in China [8]. A review showed environmental factors including geographic region, season, and climate affected vitamin C intake in five countries across Europe: Finland and neighboring Russia, India, and Mexico [9].

However, data on inadequate niacin intake trends in China from studies with large sample sizes remains limited. Further, few studies have explored the spatial distribution of the prevalence of inadequate niacin intakes in China. In this study, we used a large data set from multiple rounds of the CHNS to estimate the proportion of people with an intake less than the estimated average requirement (EAR). These data may aid in the development of interventions to reduce micronutrient deficiency and reduce the risk of diet-related diseases. We also investigated trends in the percentage of inadequate niacin intake. Further, we developed maps using Kriging spatial interpolation to provide graphical information on the prevalence of inadequate niacin intake and identify areas at high risk between 1991 and 2018 in China.

## 2. Materials and Methods

The CHNS, initiated as an ongoing longitudinal study, was conducted by the Chinese CDC and the University of North Carolina at Chapel Hill and took place in 1989, 1991, 1993, 1997, 2000, 2004, 2006, 2009, 2011, 2015, and 2018. A total of 10 rounds of the CHNS were used between 1991 and 2018. The 1989 survey was not used as it was regarded as a pretest. A multistage, cluster-randomized, stratified sampling design was employed to capture diet changes over time in a variety of areas. The surveyed area included eight provinces (Liaoning, Jiangsu, Shandong, Henan, Hubei, Hunan, Guizhou, and Guangxi) in 1991. We replaced Heilongjiang with Liaoning in 1997 and added Liaoning Province in 2000, and the number of provinces in this survey increased from eight to nine until 2000. We added three megacities (Beijing, Chongqing, and Shanghai) in 2011 and three provinces (Shaanxi, Yunnan, and Zhejiang) in 2015.

A total of two cities (the provincial capital and a lower income city) and four counties (stratified by income) were selected from each province. Within cities, two urban and two suburban communities were randomly selected. Within counties, one community in the provincial capital city and three rural villages were randomly selected. In each community, twenty households were randomly selected, and every household member was interviewed. The study design, protocol, and data collection methods were described previously [10,11]. The response rates, based on those who participated in previous survey rounds and remained in the current survey, were ~88% at the individual level and 90% at the household level [10].

The CHNS data were collected using structured questionnaires administered by field workers. The CHNS questionnaire has been used in multiple waves and shown to accurately capture income, employment, education, demographic, and diet information. All field workers were trained by nutritionists who were engaged in nutrition work or had participated in other nutritional surveys in their own counties. In each survey round, community, household, and individual data were collected. Information on dietary intake, anthropometric measurements, and sociodemographic data were collected. The interviews were carried out in the same season in each survey round. The participants signed informed consent.

### 2.1. Study Participants

The residents aged over 5 years old were included in this study because the nutrient intake data were collected from residents over 5 years of age. We excluded residents with no data on niacin intake, sex, age, education, household income, or area of residence.

### 2.2. Calculating the Intake of Niacin

The dietary data of residents was collected on three consecutive days with 24-h recalls supplemented by a daily food inventory in the family. All foods in the household, whether purchased, stored, or home-produced, were measured. We recorded and measured all food items and condiments in the home with Chinese balance scales (1991–2004) and with digital scales (2006–2018). The changes in the household food inventory and the wastage were used to estimate total household food consumption. In addition, for the 24-h recall, trained interviewers recorded the types, amounts, and places of consumption of foods. All food consumed at home or away from home was reported by residents ≥12 years of age. The parents or primary caregivers records food consumed for children <12 years of age. We calculated niacin intakes based on the compositions in the Chinese Food Composition Table. We estimated individual daily niacin intake as an average of the 3 days of dietary niacin intake.

### 2.3. Outcome Variables

The primary outcome was the prevalence of inadequate niacin intake. The consumption of food ingested by individuals was converted to niacin intake and evaluated by comparison with the age- and sex-specific EAR of Chinese residents’ dietary niacin reference intakes. The EAR is defined as the average daily nutrient intake level estimated to meet the nutrient requirement of 50% of all healthy individuals within a group. The prevalence of inadequate niacin intake was estimated using the proportion of individuals below the EAR, which represented the median niacin requirement and recommendations from the Institute of Medicine (IOM). The secondary outcome was the trend in the prevalence of inadequate niacin intake. A third outcome included the mapping of predicted high-prevalence areas; these areas were then focused on using the ordinary kriging method via ArcGIS Pro (version 2.8, Esri, Redlands, CA, USA).

### 2.4. Sociodemographic Variables

The sociodemographic variables included age, education, household income, and area of residence. The information on sociodemographic characteristics was collected from each individual (i.e., age, gender, education) or head of the household (i.e., income, area of residence). Age was categorized as 5–17, 18–44, 45–59, and ≥60 years old. Education was grouped as low (≤6 years of education), middle (7–12 years of education), and high (≥13 years of education). Areas of residence were defined as urban or rural based on population density, calculated as the total population of the community divided by the total community area obtained from official records. We used inflation-adjusted household income, which was calculated as the sum of the individual incomes of all earners in the household. The inflation-adjusted per capita household income was divided into three groups: low (<10.00 thousand yuan/per capita), moderate (≥10.00 and <20.00 thousand yuan/per capita), and high (≥20.00 thousand yuan/per capita).

### 2.5. Statistical Analysis

The means adjusted for total energy intake and the prevalence of inadequate dietary niacin intake were estimated among residents aged over 5 years old and stratified by age, sex, education, income level, and geographic area. The Chi-square test was used to compare differences among subgroups for categorical variables. In addition, general linear regression was used to calculate adjusted means and estimate trends in the adjusted prevalence of inadequate dietary niacin intake by survey year as a continuous variable. The covariates for the models included total energy intake, age, education, income, and areas of residence. Further, the sensitivity analyses were conducted in nine of the original provinces. All statistical analyses were conducted using STATA version 15 (StataCorp, College Station, TX, USA). All *p* values reported are two-tailed and we defined statistical significance as *p* < 0.05.

### 2.6. Spatial Analysis

Using Kriging spatial interpolation analysis, we estimated the prevalence of inadequate niacin intake at the county level in each round. The geographic information system (GIS) is a useful tool for displaying the distribution of diseases [12,13]. Kriging is a classical and widely used geostatistical analysis method based on the spatial autocorrelation of variables [14]. The basis of kriging is the semivariogram model, which uses the semivariance between point measures to summarize the spatial relationship. In addition, Kriging interpolation technology breaks the limitations of administrative boundaries and creates continuous surface maps. It reflects a spatial correlation that can be used to explain variation in the surface. Further, Kriging includes exploratory statistical analysis of the data, variogram modeling, creating the surface, and optionally exploring a variance surface. In recent decades, kriging has been widely used in epidemiologic studies [15,16,17,18]. The ordinary kriging is a method in which estimation is based on weighted least squares; a detailed description of this technique can be found in the literature [14].

The CHNS used survey points to collect data, which did not collect data in the whole survey county or whole survey province. The spatial interpolation makes it possible to study the overall spatial distribution trend of nutritional issues in a certain area, such as a whole county or province, from CHNS data. Based on CHNS from 1991 to 2018, we predicted the spatial distribution of the prevalence of inadequate niacin intake in a whole county and evaluated the status of inadequate niacin intake.

Due to a large amount of historical data with different codes, which was constantly updated from 1989 to 2018, we geocoded the survey sites using the unified coding method. In the 2015 round, community locations were mapped using global positioning system (GPS) coordinates. Meanwhile, the address data obtained from questionnaires was assigned longitude and latitude coordinates with ArcGIS Pro from 1991 to 2018. We geocoded all the survey sites from 1991 to 2018 using these two sources of codes in CHNS. According to the geocoding of communities obtained from GPS in 2015, the codes of communities in other rounds were checked and corrected in order to obtain more accurate geocoding. The final geocoding data was achieved for the interpolation analysis.

The niacin intake data and map data were linked to construct the geographic information database of niacin intake. We applied ordinary kriging Spatial Interpolation Analysis to calculate the prevalence of inadequate niacin intake in counties with no data by using the counties with data. We used kriging spatial interpolation analysis to create continuous surface maps of the prevalence of inadequate niacin intake and to identify significant cluster areas of inadequate niacin intake. In this study, the prevalence at each 5 km grid cell was estimated by ordinary kriging. The spatial analysis was performed using ArcGIS Pro version 2.8.

## 3. Results

### 3.1. Sample Characteristics

A total of 125,012 residents with complete dietary niacin intake data were included in this study. The mean resident age increased from 30.2 years in 1991 to 49.7 years in 2018. The proportion of older residents (aged ≥60 years) increased from 11.2% to 35.1%. The proportions for sex, urban, and rural status remained similar over time. The percentage of residents in the high-income group increased from 0.0% in 1991 to 49.5% in 2018. The percentage of residents with high education increased from 1.9% in 1991 to 11.4% in 2018 (Table 1).

### 3.2. Trends of the Prevalence of Inadequate Niacin Intake

Because the trend and magnitude of niacin intake did not change after the three megacities and three other provinces in the original cohort were joined, we utilized the results for all provinces in the statistical analysis. Overall, the prevalence of inadequate niacin intake significantly increased from 15.30% in 1991 to 28.90% in 2018. In both genders, except for the age groups 45–59 years among females, 5–17 years among males, and females with low education, significant increases in the prevalence of inadequate niacin intake were observed among age, education, house income, and area of residence subgroups between 1991 and 2018 (*p* < 0.05, Table 2). Trends of the prevalence of inadequate niacin intake was significantly increased from 13.00% in 1991 to 28.40% in 2018 for females, and from 17.75% in 1991 to 29.46% in 2018 for males.

### 3.3. Factors Associated with Inadequate Niacin Intake

In 2018, the prevalence of inadequate niacin intake was significantly higher in residents living in rural areas than in those living in urban areas; this was true for both males and females. The lowest prevalence of inadequate niacin intake was observed among females in high-income groups and among males with high-education levels. The highest prevalence of inadequate niacin intake was observed among males and females in the low-income group. Among males and females, the high-education levels were associated with the lowest prevalence of inadequate niacin intake compared with the medium- and low-education levels. There was no statistical difference in prevalence between males and females in 2018.

We conducted sensitivity analyses to identify trends in the prevalence of inadequate niacin intake in the original nine provinces. The results showed that the trends were unchanged in the original nine provinces.

### 3.4. Spatial Burden of Inadequate Niacin Intake

**Figure 1:**
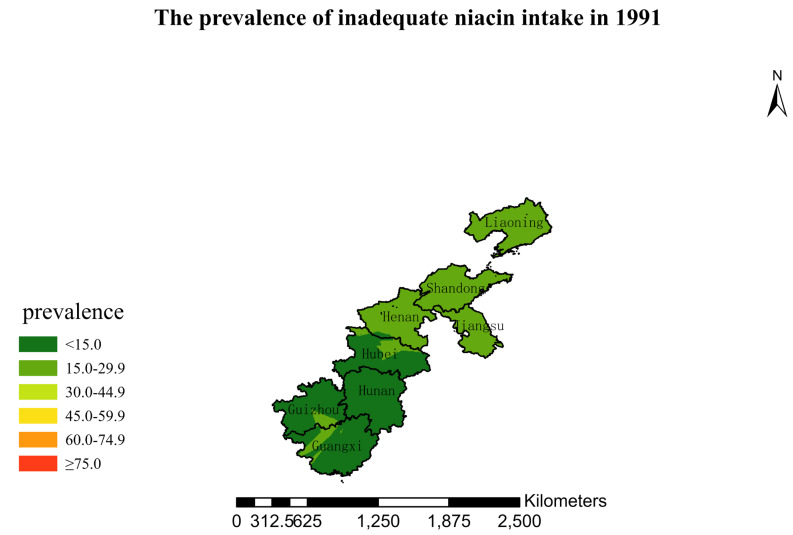
The prevalence of inadequate niacin intake by county in 1991 among Chinese Residents ≥ 5 Years of Age.

**Figure 2:**
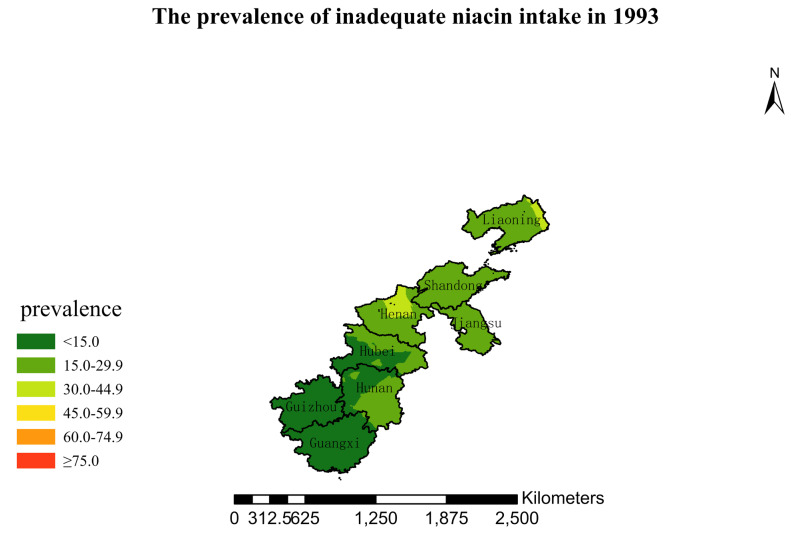
The prevalence of inadequate niacin intake by county in 1993 among Chinese Residents ≥ 5 Years of Age.

**Figure 3:**
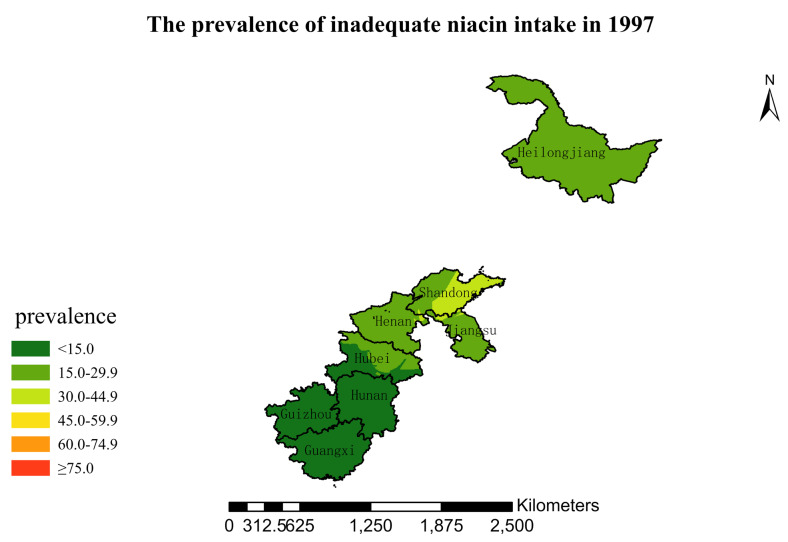
The prevalence of inadequate niacin intake by county in 1997 among Chinese Residents ≥ 5 Years of Age.

**Figure 4:**
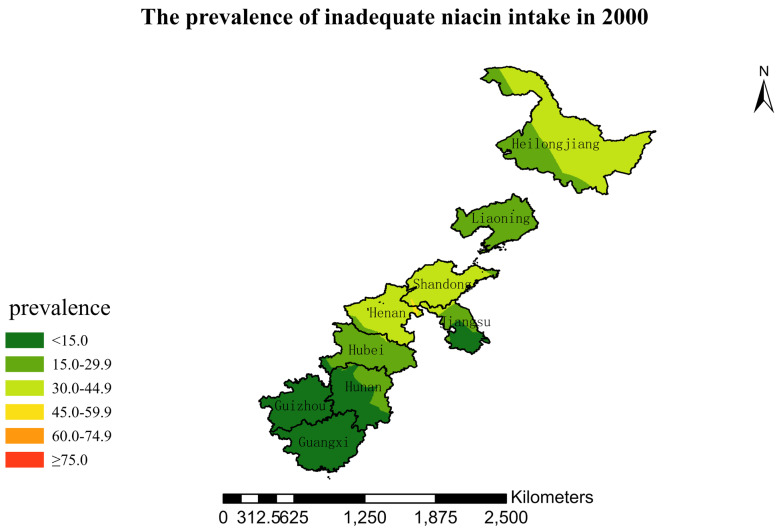
The prevalence of inadequate niacin intake by county in 2000 among Chinese Residents ≥ 5 Years of Age.

**Figure 5:**
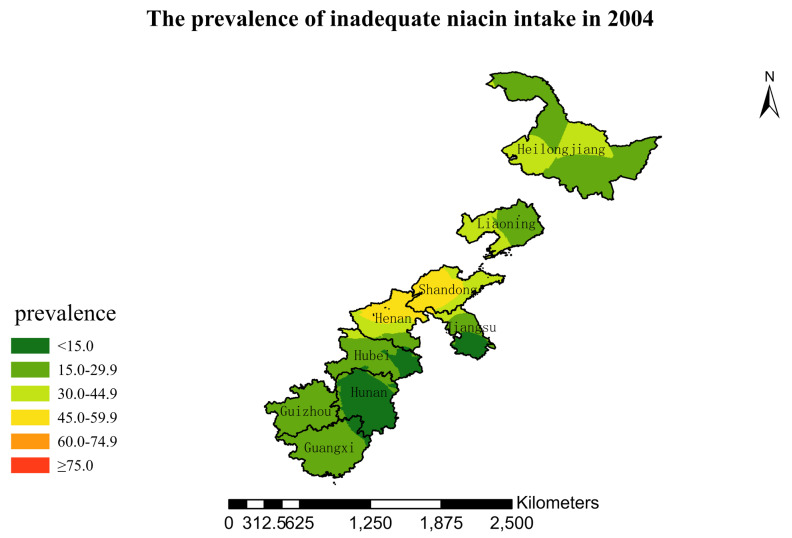
The prevalence of inadequate niacin intake by county in 2004 among Chinese Residents ≥ 5 Years of Age.

**Figure 6:**
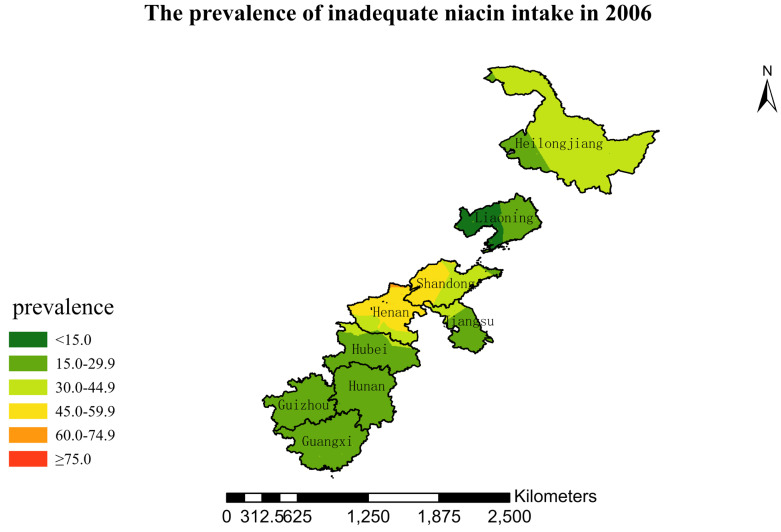
The prevalence of inadequate niacin intake by county in 2006 among Chinese Residents ≥ 5 Years of Age.

**Figure 7:**
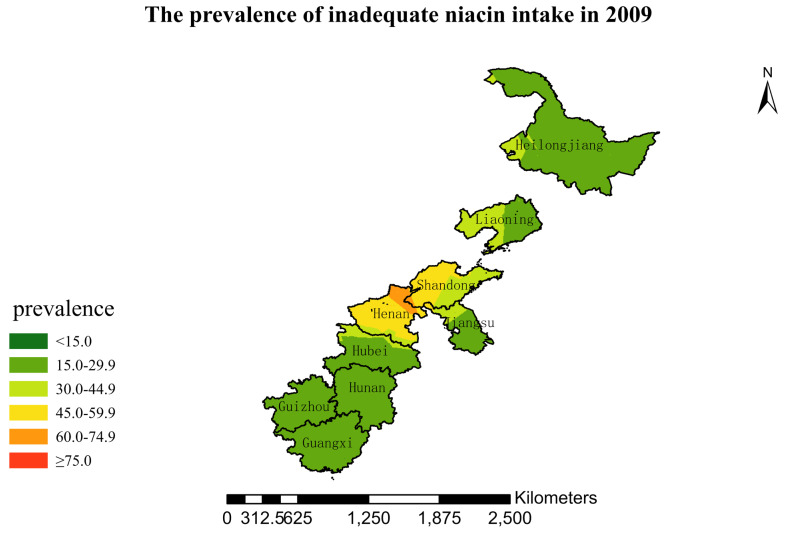
The prevalence of inadequate niacin intake by county in 2009 among Chinese Residents ≥ 5 Years of Age.

**Figure 8:**
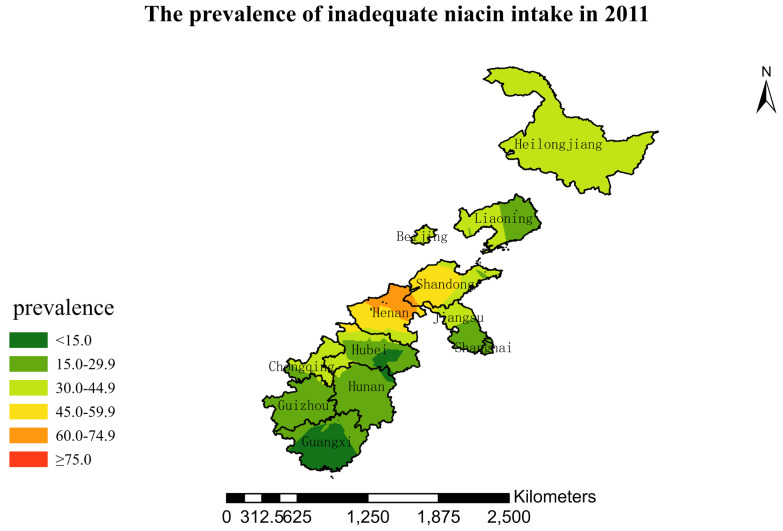
The prevalence of inadequate niacin intake by county in 2011 among Chinese Residents ≥ 5 Years of Age.

**Figure 9:**
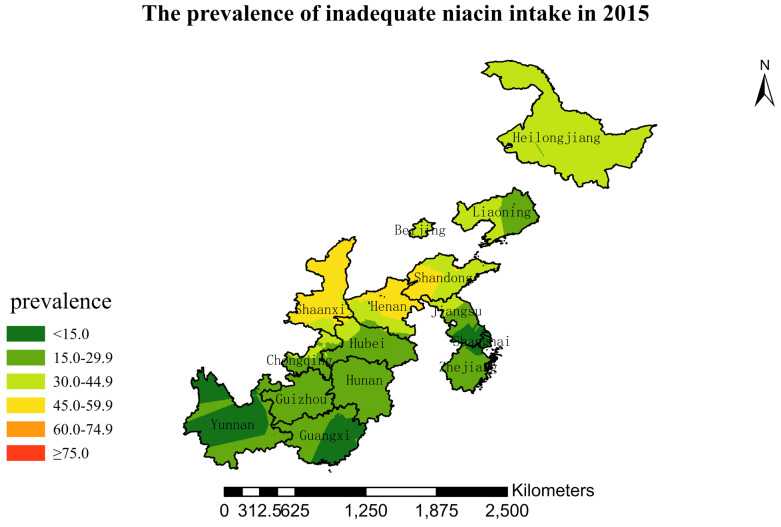
The prevalence of inadequate niacin intake by county in 2015 among Chinese Residents ≥ 5 Years of Age.

**Figure 10:**
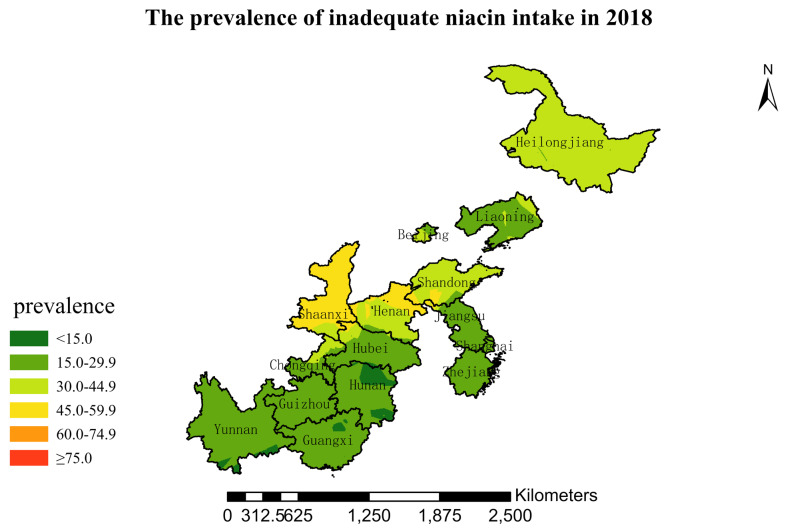
The prevalence of inadequate niacin intake by county in 2018 among Chinese Residents ≥ 5 Years of Age.

Figure 1,Figure 2,Figure 3,Figure 4,Figure 5,Figure 6,Figure 7,Figure 8,Figure 9 and Figure 10 show the geospatial distribution of the prevalence of inadequate niacin intake in the investigated provinces between 1991 and 2018. By applying Kriging spatial interpolation analysis at the county level, the estimated prevalence of inadequate niacin intake was 17.3%, 17.9%, 17.3%, 23.7%, 26.9%, 29.8%, 28.8%, 31.8%, 28.2%, and 27.50% in ten rounds.

There was a clear geographic difference in the burden of inadequate niacin intake at the provincial level in every round. In 1991, the areas with the highest prevalence of inadequate niacin intake were estimated to be the center of Henan, followed by the southeast region of Jiangsu. However, from 1993 to 2015, the highest burden of inadequate niacin intake was in Henan and Shandong. Further, twenty-seven years later (2018), the highest burden of inadequate niacin intake remained in Henan and Shandong. Notably, this prevalence has increased, not decreased, over time. In the period from 1991 to 2018, the lowest prevalence of inadequate niacin intake remained in Guangxi, Guizhou, Hunan, and Yunnan, which included the south of China.

## 4. Discussion

To our knowledge, this study is the first analysis of the deficiency of niacin intakes using GIS tools in China. The study showed that the burden of inadequate niacin intake increased among residents in China over the age of 5 between 1991 and 2018. Until 2018, 28.9% of residents over 5 years of age had a niacin intake below the EAR. Further, we found that the areas with the highest and the lowest burden of inadequate niacin intake did not change in this 27-year period.

A previous study found that the prevalence of inadequate micronutrient intake has declined in all regions, with the most pronounced decline observed in East Asia over this 50-year period [19]. However, the most surprising finding in our study was that the prevalence of inadequate niacin intake increased with the increasing availability and affordability of foods. Previous studies showed that the accessibility and availability of food increased, while our study did not include these variables. Liu et al. found that the food supply had sharply increased and the deficiency in nutrient supply had been greatly mitigated in the past five decades in China [20]. Another study documented that, due to the rapidly growing e-commerce market, food accessibility and availability in China have improved [21]. In addition, our study showed that income increased over time in China. Taken together, these data indicate that problems other than food availability and affordability were an issue in 2018. Our findings suggest that the cause of niacin deficiency in these areas is complicated.

In terms of niacin intake, there were large gaps between other countries and China. One study showed that 99.0% of the Spanish population met the European Food and Safety Authority’s recommended intakes for niacin [22]. In Germany and the UK, more than 95% of the population met the recommendations for niacin, and more than 95% of males met the recommendations for niacin in the USA [23]. The evidence from the USA also demonstrated that with enrichment, and/or fortification, and supplementation, a small percentage of the population had total usual intakes below the EAR for niacin [24]. These results suggested these interventions could be applied in China to reduce the deficiency of niacin intake.

Niacin is primarily found in plant and animal foods such as liver, kidney, lean poultry, dairy products, nuts, and leafy green vegetables. A possible reason for the findings of insufficient niacin intake among residents in this study is the cereal-based diet described in our previous study. Cereals contributed approximately 40% of niacin intake, and this was the top food source of niacin in China [25]. Moreover, the amount of coarse cereals consumed as a staple food decreased, and the consumption of refined cereals low in niacin increased in China [26,27]. The traditional cooking practices, including the process of cleaning and cooking, may also explain the insufficient intake of niacin in China [28]. Moreover, high-yield cereals have replaced more nutrient-rich cereals [29]. Another explanation may be that fortification, which has decreased the prevalence of inadequate micronutrient intakes in all regions worldwide, has not been applied on a large scale in China. Additionally, only 0.71% of residents used nutrient supplements between 2010 and 2012, according to the China Nutrition and Health Surveillance study [30].

The geospatial analysis showed that the highest and lowest burdens of inadequate intakes of niacin appeared unchanged; Henan, Shandong, and Shaanxi had the highest burden, and Guangxi, Hunan, and Yunnan had the lowest burden. The results suggest a stable distribution of epidemiologic profiles of niacin intake at the county and provincial levels over the 27-year interval. This may be explained by local eating habits and food supplies. Henan and Shandong are agricultural areas with a tradition of cultivating and consuming wheat and other cereals. According to the China Food Composition Table, the niacin content of wheat and rice is 4 mg/100 g and 2 mg/100 g, respectively. The wheat yield was among the highest in China, and wheat was the staple food in these two provinces. On the contrary, the staple food was rice in Guangxi, Guizhou, and Yunnan (located in the south and southwest of China). If only staple food were taken into account, Guangxi, Guizhou, and Yunnan should have the highest burden of inadequate intakes of niacin, not Henan, Shandong, and Shaanxi. The truth was the opposite. Therefore, the geographical disparities in inadequate intakes of niacin may be due to food diversity. The south and southwest of China have an abundance of fruits, vegetables, and poultry and a higher diversity of foods than other provinces. In addition to the reasons mentioned above, more studies are needed to further identify the causes of niacin deficiency.

Our findings showed that low-income status was associated with the highest prevalence of inadequate niacin intake between both sexes. Previous studies demonstrated that micronutrient deficiencies are found more often in the lower and lower-middle income populations or countries, consistent with our results [31,32,33]. The evidence showed that higher education and income were associated with higher scores for higher-quality diets [34]. These results emphasize the need for interventions for these disadvantaged subpopulations.

Dietary intake is the primary source of niacin in China, and the main strategy for controlling nutrient deficiencies is focusing on dietary diversity. The strategies recommended by the World Health Organization (WHO) and the United Nations Food and Agriculture Organization (FAO) for the control of micronutrient malnutrition include complementary approaches such as dietary diversification, nutrition education, food fortification, public health and food safety measures, and supplementation [35]. The geographic location may no longer be a barrier to achieving dietary diversity for rural or urban populations due to the rapid development of the e-commerce market and online-to-offline food delivery in China [21,36]. Therefore, nutrition education is more important and necessary in the highest-burden areas of China. Furthermore, for higher-risk residents, supplementation and food fortification are also effective choices [37].

This study provides information to guide health policy and program implementation in China. Provinces with the highest burden of inadequate niacin intake should be a priority for policy intervention. Previous studies have found that the dietary knowledge level of most rural residents in China is low [38,39]. Our results show that residents living in the suburbs had a higher prevalence of inadequate niacin intake in 2018 compared to those living in urban areas. This contrasts with the data from 1991, which showed that niacin deficiency rates were higher in urban than rural areas. This indicates that niacin intake changed over time in these areas. Therefore, target groups for nutrient education programs should be the residents living in the suburbs of Henan, Shandong, and Shaanxi.

Our study has several strengths. This is the first attempt to quantify the burden of insufficient niacin intake by applying the geographic information system method. In addition, we used data from the largest and longest study of its kind in China, which enabled us to examine trends across 27 years. We applied Kriging’s interpolation method to visually explore and estimate prevalence in counties without data to obtain a complete image for niacin deficiency control. The spatial visualization of nutrient deficiency collects data according to administrative divisions, and uses different colors or patterns to display nutrient deficiency levels in different regions. Intuitively describing the characteristics of nutrient deficiency in different regions and revealing spatial distribution trends provide clues for further nutrient deficiency research and control programs. The spatial distribution of nutrient deficiency is the basis for subsequent spatial analysis and modeling. Because traditional space mapping is restricted by the borders of a county, city, or province, the distribution of niacin deficiency is not continuous and smooth. Meanwhile, regionalized variables ignore some potential relation. The ordinary kriging spatial interpolation method, which we applied in this study, broke the limitation of the administrative region of the terrain map and more scientifically revealed the spatial distribution pattern of niacin deficiency. Due to its simplicity and accuracy, ordinary kriging is the most general and widely used interpolation method in geostatistics. It weights the surrounding measured values to obtain a linear prediction system of unmeasured positions and provides estimation errors with high interpolation accuracy. This study also had limitations. We did not collect data on supplement intake. However, studies have shown that the percentage of residents using nutrient supplements is very low, as mentioned above.

## 5. Conclusions

In this study it was observed that the prevalence of inadequate niacin intake in China increased over time and varied by geography. The findings identify areas in China that should be targeted for intervention. Specifically, the regions with the highest prevalence of inadequate niacin intakes include Henan and Shandong. Therefore, interventions should focus on those areas. Addressing niacin intake in China will require the tailoring of effective programs to address the needs of different geographical areas.

## Figures and Tables

**Table 1:** Characteristics of Chinese residents aged over 5 years by CHNS, 1991–2018.

	1991 (*n* = 11,011)	1993 (*n* = 10,420)	1997 (*n* = 10,414)	2000 (*n* = 10,191)	2004 (*n* = 10,819)	2006 (*n* = 10,399)	2009 (*n* = 10,668)	2011 (*n* = 14,143)	2015 (*n* = 17,092)	2018 (*n* = 14,547)
Age (years)										
5–17	2706 (24.6)	2563 (24.6)	2353 (22.6)	1846 (18.1)	1552 (14.4)	1255 (12.1)	1135 (10.6)	1555 (11.0)	2038 (11.9)	1629 (11.2)
18–44	5267 (47.8)	4787 (45.9)	4497 (43.2)	4149 (40.7)	4023 (37.2)	3783 (36.4)	3645 (34.2)	4471 (31.6)	5056 (29.6)	3464 (23.8)
45–59	1810 (16.4)	1800 (17.3)	2148 (20.6)	2615 (25.7)	3175 (29.3)	3099 (29.8)	3334 (31.3)	4497 (31.8)	5071 (29.7)	4344 (29.9)
≥60	1228 (11.2)	1270 (12.2)	1416 (13.6)	1581 (15.5)	2069 (19.1)	2262 (21.8)	2554 (23.9)	3620 (25.6)	4927 (28.8)	5110 (35.1)
Sex										
Men	5336 (48.5)	5098 (48.9)	5186 (49.8)	5161 (50.6)	5240 (48.4)	5033 (48.4)	5216 (48.9)	6718 (47.5)	8137 (47.6)	6878 (47.3)
Women	5675 (51.5)	5322 (51.1)	5228 (50.2)	5030 (49.4)	5579 (51.6)	5366 (51.6)	5452 (51.1)	7425 (52.5)	8955 (52.4)	7669 (52.7)
Education										
Low	6470 (58.8)	5798 (55.6)	5752 (55.2)	4943 (48.5)	4750 (43.9)	4532 (43.6)	4482 (42.0)	5191 (36.7)	6050 (35.4)	4895 (33.7)
Medium	4332 (39.3)	4412 (42.4)	4442 (42.7)	4930 (48.4)	5604 (51.8)	5264 (50.6)	5601 (52.5)	7635 (54.0)	9241 (54.1)	7992 (54.9)
High	209 (1.9)	210 (2.0)	220 (2.1)	318 (3.1)	465 (4.3)	603 (5.8)	585 (5.5)	1317 (9.3)	1801 (10.5)	1660 (11.4)
Income (1000 yuan/per capita) ^1^										
Low	11,011 (100.0)	10,409 (99.9)	10,274 (98.7)	9798 (96.1)	9552 (88.3)	8710 (83.8)	7172 (67.2)	6764 (47.8)	6238 (36.5)	4125 (28.4)
Medium	0 (0.0)	11 (0.1)	125 (1.2)	315 (3.1)	1014 (9.4)	1309 (12.6)	2450 (23.0)	4144 (29.3)	4404 (25.8)	3209 (22.1)
High	0 (0.0)	0 (0.0)	15 (0.1)	78 (0.8)	253 (2.3)	380 (3.7)	1046 (9.8)	3235 (22.9)	6450 (37.7)	7213 (49.5)
Urban/rural										
Urban	7466 (67.8)	7323 (70.3)	7485 (71.9)	7377 (72.4)	7495 (69.3)	7211 (69.3)	7397 (69.3)	8230 (58.2)	10,334 (60.5)	9080 (62.4)
Rural	3545 (32.2)	3097 (29.7)	2929 (28.1)	2814 (27.6)	3324 (30.7)	3188 (30.7)	3271 (30.7)	5913 (41.8)	6758 (39.5)	5467 (37.6)

^1^ Per capita household income adjusted by consumer price index to 2018 Chinses yuan value.

**Table 2:** Trends in dietary intake of niacin among residents aged ≥5 years, the China Health and Nutrition Survey (CHNS) 1991–2018.

	1991 (*n* = 11,011)		1993 (*n* = 10,420)		1997 (*n* = 10,414)		2000 (*n* = 10,191)		2004 (*n* = 10,819)		2006 (*n* = 10,399)		2009 (*n* = 10,668)		2011 (*n* = 14,143)		2015 (*n* = 17,092)		2018 (14,030)		ADJUSTED Model ^1^
	*n*	<EAR%	*n*	<EAR%	*n*	<EAR%	*n*	<EAR%	*n*	<EAR%	*n*	<EAR%	*n*	<EAR%	*n*	<EAR%	*n*	<EAR%	*n*	<EAR%	*p*-Value for Trend ^2^
Total	11,011	15.3	10,420	17.74	10,414	16.67	10,191	23.35	10,819	28.11	10,399	29.66	10,668	29.31	14,143	31.76	17,092	27.99	14,547	28.9	0.001
Women	5675	13	5322	15.01	5228	15.23	5030	22.09	5579	27.21	5366	28.66	5452	28.93	7425	32.36	8955	27.52	7669	28.4	0.001
Age																					
5–17	1338	17.79	1232	19.4	1115	22.6	840	31.55	734	35.97	578	39.1	502	42.83	759	35.7	958	31.42	754	31.83	0.001
18–44	2781	9.78	2493	12.64	2283	10.73	2017	17.7	2095	22.96	1991	25.57	1886	25.08	2411	30.61	2765	24.23	1916	28.03	0.005
45–59	919	12.4	930	11.72	1087	12.7	1328	18.9	1663	25.26	1604	24.5	1717	26.44	2356	29.24	2643	26.86	2294	26.2	0.359
≥60	637	17.9	667	20.39	743	21.67	845	28.17	1087	32.47	1193	34.37	1347	32.29	1899	37.12	2589	30.24	2705	29.57	0.001
Education																					
Low	3640	12.55	3297	14.92	3254	15.55	2846	22.91	2865	28.24	2715	30.09	2673	29.7	3151	34.72	3593	29.45	2959	30.92	0.086
Medium	1973	13.68	1965	15.22	1908	14.73	2085	21.06	2540	26.22	2412	27.94	,2537	28.54	3667	30.87	4496	27.22	3892	27.08	0.005
High	62	17.74	60	13.33	66	13.64	99	20.2	174	24.71	239	19.67	242	24.38	607	29.16	866	21.02	818	25.55	0.001
Income (1000 yuan/per capita) ^3^																					
Low	5675	13	5318	15.01	5158	15.32	4835	22.21	4929	27.92	4524	29.66	3714	30.86	3661	36.85	3351	32.14	2214	32.66	0.001
Medium	0	0	4	25	65	9.23	160	16.88	521	21.31	655	25.04	1219	25.18	2119	28.98	2287	28.03	1674	30.47	0.001
High	0	0	0	0	5	0	35	28.57	129	24.03	187	17.11	519	23.89	1645	26.75	3317	22.49	3781	24.99	0.001
Urban/rural																					
Urban	1821	14.61	1591	16.47	1513	15.27	1422	22.36	1754	27.42	1666	29.05	1686	30.31	3122	29.98	3562	24.82	2918	25.77	0.001
Rural	3854	12.25	3731	14.39	3715	15.21	3608	21.98	3825	27.11	3700	28.49	3766	28.31	4303	34.09	5393	29.3	4751	30.01	0.001
Men	5336	17.75	5098	20.58	5186	18.13	5161	24.59	5240	29.06	5033	30.72	5216	29.72	6718	31.1	8137	28.51	6878	29.46	0.001
Age																					
5–17	1368	23.9	1331	24.42	1238	27.38	1006	34.1	818	38.51	677	42.54	633	39.34	796	37.56	1080	34.17	875	33.94	0.062
18–44	2486	13.52	2294	17.18	2214	11.74	2132	18.11	1928	24.38	1792	24.83	1759	23.93	2060	24.27	2291	22.61	1548	24.61	0.001
45–59	891	16.72	870	19.08	1061	14.7	1287	21.37	1512	26.79	1495	26.02	1617	27.4	2141	29.94	2428	25.95	2050	27.85	0.001
≥60	591	22.84	603	27.2	673	27.49	736	36.01	982	33.91	1069	39.66	1207	36.21	1721	37.71	2338	34.35	2405	32.31	0.001
Education																					
Low	2830	19.29	2501	21.35	2498	19.5	2097	28.47	1885	30.66	1817	36.21	1809	33.33	2040	36.81	2457	32.72	1936	32.85	0.001
Medium	2359	16.11	2447	19.98	2534	17.05	2845	22.04	3064	28.56	2852	28.16	3064	28.2	3968	29.13	4745	27.71	4100	28.83	0.001
High	147	14.29	150	17.33	154	13.64	219	20.55	291	24.05	364	23.35	343	24.2	710	25.63	935	21.5	842	24.7	0.001
Income (1000 yuan/per capita) ^3^																					
Low	5336	17.75	5091	20.6	5116	18.26	4963	24.76	4623	30.18	4186	31.96	3458	32.01	3103	36	2887	35.19	1911	35.58	0.001
Medium	0	0	7	0	60	10	155	20	493	21.3	654	27.83	1231	26.24	2025	26.52	2117	27.16	1535	30.36	0.001
High	0	0	0	0	10	0	43	20.93	124	18.55	193	13.47	527	22.77	1590	27.36	3133	23.27	3432	25.64	0.001
Urban/rural																					
Urban	1724	17.81	1506	20.72	1416	18.93	1392	23.71	1570	27.71	1522	31.54	1585	31.61	2791	29.85	3196	26.41	2549	27.62	0.001
Rural	3612	17.72	3592	20.52	3770	17.82	3769	24.91	3670	29.65	3511	30.36	3631	28.89	3927	31.98	4941	29.87	4329	30.54	0.033

^1^ Adjusted for total energy intake. ^2^ *p*-value from the linear regression by modeling the survey period as a continuous variable. ^3^ Per capita household income adjusted by consumer price index to 2018 Chinees yuan value

## Data Availability

Not applicable.

## References

[1] Zhang J., Song P.K., Zhao L.Y., Sun Y., Yu K., Yin J., Pang S.J., Liu Z., Man Q.Q., He L. (2021). Malnutrition in Relation with Dietary, Geographical, and Socioeconomic Factors among Older Chinese. Biomed. Environ. Sci..

[2] Jenkins D.J.A., Spence J.D., Giovannucci E.L., Kim Y.I., Josse R., Vieth R., Blanco Mejia S., Viguiliouk E., Nishi S., Sahye-Pudaruth S. (2018). Supplemental Vitamins and Minerals for CVD Prevention and Treatment. J. Am. Coll. Cardiol..

[3] Lavigne P.M., Karas R.H. (2013). The current state of niacin in cardiovascular disease prevention: A systematic review and meta-regression. J. Am. Coll. Cardiol..

[4] Gasperi V., Sibilano M., Savini I., Catani M.V. (2019). Niacin in the Central Nervous System: An Update of Biological Aspects and Clinical Applications. Int. J. Mol. Sci..

[5] Zhang Z., Liu M., Zhou C., He P., Zhang Y., Li H., Li Q., Liu C., Qin X. (2021). Evaluation of Dietary Niacin and New-Onset Hypertension Among Chinese Adults. JAMA Netw. Open.

[6] Wu Y., Li S., Wang W., Zhang D. (2020). Associations of dietary vitamin B1, vitamin B2, niacin, vitamin B6, vitamin B12 and folate equivalent intakes with metabolic syndrome. Int. J. Food. Sci. Nutr..

[7] Newby P.K., Noel S.E., Grant R., Judd S., Shikany J.M., Ard J. (2012). Race and region have independent and synergistic effects on dietary intakes in black and white women. Nutr. J..

[8] Du S., Wang H., Zhang B., Popkin B.M. (2020). Dietary Potassium Intake Remains Low and Sodium Intake Remains High, and Most Sodium is Derived from Home Food Preparation for Chinese Adults, 1991-2015 Trends. J. Nutr..

[9] Carr A.C., Rowe S. (2020). Factors Affecting Vitamin C Status and Prevalence of Deficiency: A Global Health Perspective. Nutrients.

[10] Popkin B.M., Du S., Zhai F., Zhang B. (2010). Cohort Profile: The China Health and Nutrition Survey--monitoring and understanding socio-economic and health change in China, 1989-2011. Int. J. Epidemiol..

[11] Zhang B., Zhai F.Y., Du S.F., Popkin B.M. (2014). The China Health and Nutrition Survey, 1989-2011. Obes. Rev..

[12] Kirby R.S., Delmelle E., Eberth J.M. (2017). Advances in spatial epidemiology and geographic information systems. Ann. Epidemiol..

[13] Banerjee S. (2016). Spatial Data Analysis. Annu. Rev. Public Health.

[14] Wackernagel H. (1995). Ordinary Kriging. Multivariate Geostatistics: An Introduction with Applications.

[15] Huang S., Xiang H., Yang W., Zhu Z., Tian L., Deng S., Zhang T., Lu Y., Liu F., Li X. (2020). Short-term Effect of Air Pollution on Tuberculosis Based on Kriged Data: A Time-series Analysis. Int. J. Environ. Res. Public Health.

[16] Patil B., Hegde V., Sridhara S., Narayanaswamy H., Naik M.K., Patil K.K.R., Rajashekara H., Mishra A.K. (2022). Exploring the Impact of Climatic Variables on Arecanut Fruit Rot Epidemic by Understanding the Disease Dynamics in Relation to Space and Time. J. Fungi.

[17] Liyew A.M., Sisay M.M., Muche A.A. (2021). Spatial distribution and factors associated with low birth weight in Ethiopia using data from Ethiopian Demographic and Health Survey 2016: Spatial and multilevel analysis. BMJ Paediatr. Open.

[18] Seboka B.T., Hailegebreal S., Mamo T.T., Yehualashet D.E., Gilano G., Kabthymer R.H., Ewune H.A., Kassa R., Debisa M.A., Yawo M.N. (2022). Spatial trends and projections of chronic malnutrition among children under 5 years of age in Ethiopia from 2011 to 2019: A geographically weighted regression analysis. J. Health Popul. Nutr..

[19] Beal T., Massiot E., Arsenault J.E., Smith M.R., Hijmans R.J. (2017). Global trends in dietary micronutrient supplies and estimated prevalence of inadequate intakes. PLoS ONE.

[20] Liu A., Han A., Chai L. (2021). Assessing the Nutrient Adequacy in China's Food Supply from 1965 to 2018. Nutrients.

[21] Maimaiti M., Zhao X., Jia M., Ru Y., Zhu S. (2018). How we eat determines what we become: Opportunities and challenges brought by food delivery industry in a changing world in China. Eur. J. Clin. Nutr..

[22] Mielgo-Ayuso J., Aparicio-Ugarriza R., Olza J., Aranceta-Bartrina J., Gil A., Ortega R.M., Serra-Majem L., Varela-Moreiras G., Gonzalez-Gross M. (2018). Dietary Intake and Food Sources of Niacin, Riboflavin, Thiamin and Vitamin B(6) in a Representative Sample of the Spanish Population. The Anthropometry, Intake, and Energy Balance in Spain (ANIBES) Study dagger. Nutrients.

[23] Troesch B., Hoeft B., McBurney M., Eggersdorfer M., Weber P. (2012). Dietary surveys indicate vitamin intakes below recommendations are common in representative Western countries. Br. J. Nutr..

[24] Fulgoni V.L., Keast D.R., Bailey R.L., Dwyer J. (2011). Foods, fortificants, and supplements: Where do Americans get their nutrients?. J. Nutr..

[25] Li L., Zhang B., Wang H.J., Ouyang Y.F., Huang F.F., Wang Y., Zhang J.G., Su C., Du W.W., Jia X.F. (2020). Sociodemographic Factors Associated with Dietary Intake of Thiamine, Riboflavin, and Niacin among Chinese Adults in 2015. Biomed. Environ. Sci..

[26] Chang X., DeFries R.S., Liu L., Davis K. (2018). Understanding dietary and staple food transitions in China from multiple scales. PLoS ONE.

[27] Yu D., Zhao L., Zhao W. (2020). Status and trends in consumption of grains and dietary fiber among Chinese adults (1982-2015). Nutr. Rev..

[28] Society C.N. (2016). The Dietary Guidelines for Chinese Residents (2016).

[29] DeFries R., Fanzo J., Remans R., Palm C., Wood S., Anderman T.L. (2015). Global nutrition. Metrics for land-scarce agriculture. Science.

[30] Gong W., Liu A., Yao Y., Ma Y., Ding C., Song C., Yuan F., Zhang Y., Feng G., Chen Z. (2018). Nutrient Supplement Use among the Chinese Population: A Cross-Sectional Study of the 2010(-)2012 China Nutrition and Health Surveillance. Nutrients.

[31] Mark H.E., Houghton L.A., Gibson R.S., Monterrosa E., Kraemer K. (2016). Estimating dietary micronutrient supply and the prevalence of inadequate intakes from national Food Balance Sheets in the South Asia regiona. Asia Pac. J. Clin. Nutr..

[32] Torheim L.E., Ferguson E.L., Penrose K., Arimond M. (2010). Women in resource-poor settings are at risk of inadequate intakes of multiple micronutrients. J. Nutr..

[33] Barennes H., Sengkhamyong K., Rene J.P., Phimmasane M. (2015). Beriberi (thiamine deficiency) and high infant mortality in northern Laos. PLoS Negl. Trop. Dis..

[34] Mullie P., Clarys P., Hulens M., Vansant G. (2010). Dietary patterns and socioeconomic position. Eur. J. Clin. Nutr..

[35] WHO, FAO (2006). Guidelines on Food Fortification with Micronutrients.

[36] Zhao X., Lin W., Cen S., Zhu H., Duan M., Li W., Zhu S. (2021). The online-to-offline (O2O) food delivery industry and its recent development in China. Eur. J. Clin. Nutr..

[37] Dwyer J.T., Wiemer K.L., Dary O., Keen C.L., King J.C., Miller K.B., Philbert M.A., Tarasuk V., Taylor C.L., Gaine P.C. (2015). Fortification and health: Challenges and opportunities. Adv. Nutr..

[38] Hou M., Qing P., Min S. (2021). Multiple indicators of household dietary diversity in rural China: Effects of income and dietary knowledge. Nutrition.

[39] Cui B., Wang L.D., Wang F.R., Peng J., Ma J.Y., Chen X., Xu M.Y., Ke J., Tian Y. (2022). Correlation between dietary information sources and knowledge of adequate diets in Eastern China. Front. Public Health.

